# Mechanisms of bioleaching: iron and sulfur oxidation by acidophilic microorganisms

**DOI:** 10.1042/EBC20220257

**Published:** 2023-08-11

**Authors:** Sarah Jones, Joanne M. Santini

**Affiliations:** 1 Institute of Structural and Molecular Biology, Division of Biosciences, University College London, WC1E 6BT, U.K.; 2 Institute of Structural and Molecular Biology, Division of Biosciences, Birkbeck, University of London, Malet Street, London, WC1E 7HX, U.K.

**Keywords:** Acidophiles, Bioleaching, Iron oxidation, Sulfur oxidation

## Abstract

Bioleaching offers a low-input method of extracting valuable metals from sulfide minerals, which works by exploiting the sulfur and iron metabolisms of microorganisms to break down the ore. Bioleaching microbes generate energy by oxidising iron and/or sulfur, consequently generating oxidants that attack sulfide mineral surfaces, releasing target metals. As sulfuric acid is generated during the process, bioleaching organisms are typically acidophiles, and indeed the technique is based on natural processes that occur at acid mine drainage sites. While the overall concept of bioleaching appears straightforward, a series of enzymes is required to mediate the complex sulfur oxidation process. This review explores the mechanisms underlying bioleaching, summarising current knowledge on the enzymes driving microbial sulfur and iron oxidation in acidophiles. Up-to-date models are provided of the two mineral-defined pathways of sulfide mineral bioleaching: the thiosulfate and the polysulfide pathway.

## Introduction

Bioleaching is a technique that uses microorganisms to remove metals from ore where traditional extraction methods are not economically viable. This technique is commonly used for sulfide mineral ores, which are the source of numerous valuable metals such as gold, silver and copper. As well as being energy intensive and polluting, traditional methods of metal extraction from sulfide minerals (e.g. pyrometallurgy) are expensive [1]. Therefore, low quality ores are typically not processed using these techniques, and may be discarded as waste. Bioleaching offers a cost-effective and low input solution to this problem, by exploiting microbial metabolisms to break metal ores down.

During the bioleaching process, microbes generate energy by oxidising sulfur and iron from sulfide minerals. The resulting oxidants attack the sulfide minerals, leading to the release of target metals. While the overall concept of bioleaching is simple, the underlying mechanisms are complex; microbial iron and sulfur metabolisms rely on a complex series of enzymes to facilitate the process.

In this review, we summarise what is currently known regarding the overall mechanisms of bioleaching, including an overview of the genes driving aerobic iron and sulfur oxidation. Two common bioleaching substrates, chalcopyrite and pyrite, are used to model theoretical bioleaching pathways. Due to the typically acidic conditions in which bioleaching takes place, the focus of this review is on the bioleaching mechanisms of acidophilic microorganisms.

## Bioleaching: an overview of the key concepts

**Figure 1:**
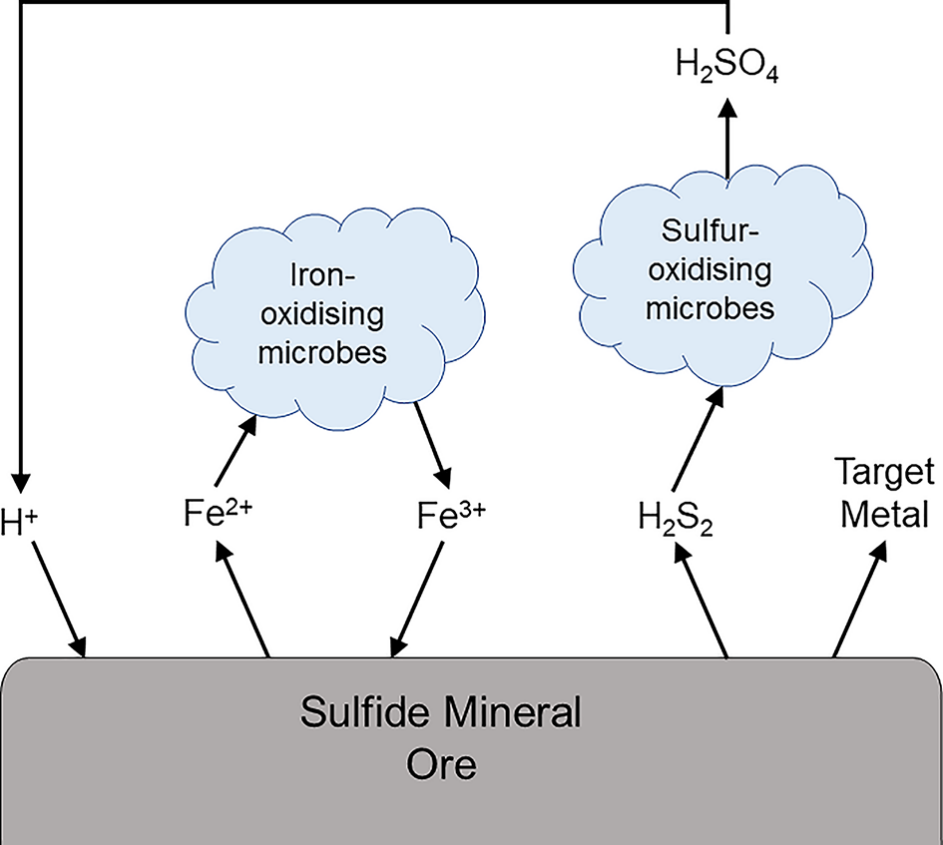
Simplified overview of the bioleaching process showing the regeneration of oxidants by iron- and sulfur- oxidising microbes, resulting in the release of target metals

Bioleaching is based on microbially driven sulfur and iron oxidation processes that naturally occur in the waters of former mine sites (acid mine drainage). The metabolisms of iron- and sulfur- oxidising bacteria enhance the breakdown of sulfide minerals through the regeneration of protons (via sulfuric acid) and the oxidant Fe^3+^, respectively (Figure 1). Protons and Fe^3+^ ‘attack’ the mineral, breaking the chemical bonds holding the sulfide anion to the metal(s) within the mineral structure. This leads to the release of the target metal(s).

Due to the production of sulfuric acid resulting from sulfide oxidation to sulfate [2], and the low nutrient environment of ‘bare’ mineral substrates [3], the organisms used for bioleaching are typically acidophilic chemolithoautotrophs. These are microbes that thrive in low-pH environments, capable of exploiting inorganic electron sources (e.g*.* sulfur and iron) for energy generation and receiving carbon through CO_2_ fixation. Heterotrophs, on the other hand, are typically regarded as only playing an indirect role in mineral dissolution, at a community level, by metabolising organic compounds that can inhibit chemolithotrophic activity [4]. Many different acidophilic species have been successfully used in bioleaching applications. The diversity of microbes identified in bioleaching systems has been covered by a number of other reviews [5–7].

### Direct versus indirect dissolution of sulfide minerals

Much of the early literature regarding bioleaching describes the potential existence of a ‘direct’ mechanism of sulfide mineral oxidation whereby microbes attach to the mineral surface and directly oxidise sulfide without ferric iron as an oxidant [8,9]. It has now been widely acknowledged that this mechanism is unlikely to exist [10–12]. Instead, reduced sulfur is released from the mineral structure following proton or Fe^3+^ attack.

Although no direct oxidation of sulfur takes place within the mineral structure, it has been proposed that there may be contact and non-contact bioleaching. The former describes leaching that occurs via cells attached to the mineral surface (within an EPS matrix) generating ferric iron, and the latter defined as bioleaching facilitated by planktonic microorganisms oxidising iron which then oxidises sulfur when it encounters mineral surfaces [13]. An additional process of ‘cooperative leaching’ has also been described, whereby some free-living bacteria oxidise sulfur species released by contact leaching bacteria [14]. In this instance, contact and indirect bioleaching processes occur concurrently [15].

## Biochemistry of microbial sulfur oxidation

In the 136 years since the discovery of the first sulfur-oxidising microbe, *Beggiatoa* [16], a great deal of knowledge has been acquired regarding the biochemical mechanisms of sulfur oxidation processes. Nonetheless, due to the complexity of the biochemistry involved and the diversity of species capable of sulfur oxidation, much also remains unknown.

There are a large number of enzymes and proteins that have the potential to catalyse the oxidation of reduced inorganic sulfur compounds (RISCs) [17]. There is often more than one catalyst for each RISC, and the number of sulfur oxidation pathways is almost as great as the diversity of microbes capable of oxidative sulfur metabolism.

**Figure 2:**
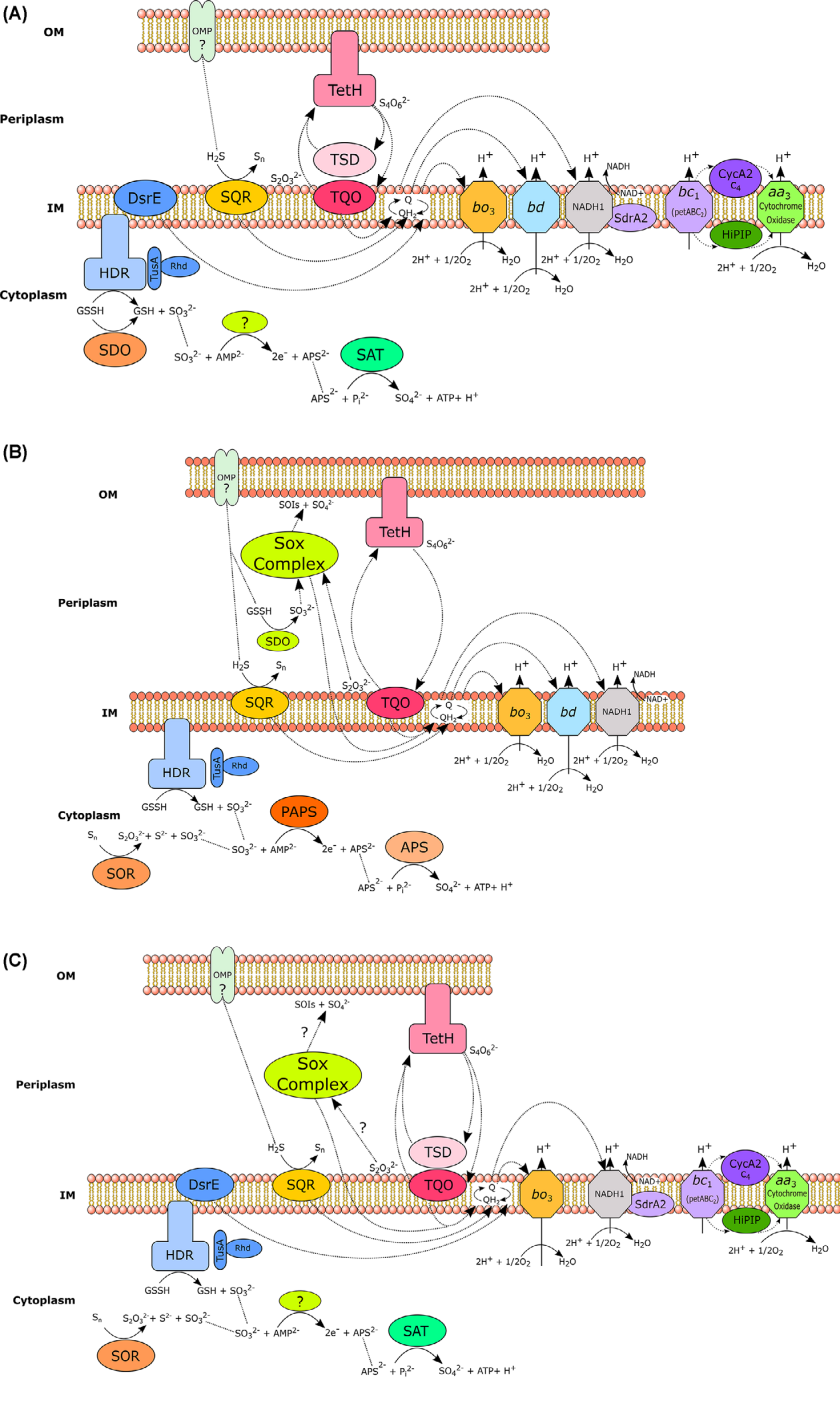
Model of sulfur oxidation in the *Acidithiobacilli* Model of sulfur oxidation in (**A**) *A. ferrooxidans*; (**B**) *A. thiooxidans* and (**C**) *A. ferrivorans*. Sulfide oxidation proceeds via the inner membrane-bound sulfide-quinone reductase (SQR), which facilitates the oxidation of hydrogen sulfide to elemental sulfur. Insoluble elemental sulfur in the periplasm is most likely converted to glutathionate persulfide (GSSH) by membrane bound thiols prior to oxidation. This GSSH is transported via transferases (DsrE, TusA and Rhd) to a heterodisulfide reductase (HDR) complex, which catalyses its oxidation to sulfite and GSH. Alternatively, elemental sulfur may be oxidised by sulfur oxygenase reductase (SOR) or sulfur dioxygenase (SDO). It is predicted that sulfite oxidation in *A. ferrooxidans* and *A. ferrivorans* is catalysed by an as-yet unknown enzyme, generating adenosine-5*′*-phosphosulfate (APS), which is then further oxidised to sulfate, with concomitant ATP and proton generation by sulfate adenylyltransferase (SAT). In A. thiooxidans, sulfite oxidation occurs via phosphoadenosine phosphosulfate (PAPS) reductase, where sulfite is first oxidised to PAPS by the PAPS reductase, then oxidised to APS, and sulfate by APS kinase. In all three species, the oxidation of thiosulfate to tetrathionate is mediated by thiosulfate quinone oxidoreductase (TQO) or thiosulfate dehydrogenase (TSD), while an outer membrane-bound, homodimeric tetrathionate hydrolase (TetH) hydrolyses tetrathionate to thiosulfate. *A. thiooxidans* and *A. ferrivorans* both possess the alternative sulfur oxidation pathway, SOX. Across the Acidithiobacilli, electrons produced by RISC oxidation are thought to be transferred to the quinone pool (Q/QH_2_), from which they are transported to the membrane bound terminal oxidases *bo*_3_ and/or *bd*. Alternately, the electrons generated in RISC oxidation can be transferred indirectly to an *aa*_3_ oxidase for O_2_ reduction (via high potential iron-sulfur protein (HiPIP)), or to a NADH_1_ complex, via which NADH can be generated. These figures were created based on information collected [16–30].

As the best studied genus of acidophiles, it is unsurprising that *Acidithiobacillus* presents some of the most complete models of sulfur oxidation. Acidithiobacilli can facilitate the complete aerobic oxidation of sulfide to sulfate, following a series of oxidation steps mediated by an array of enzymes and proteins, as outlined in Figure 2A–C.

**Figure 3:**
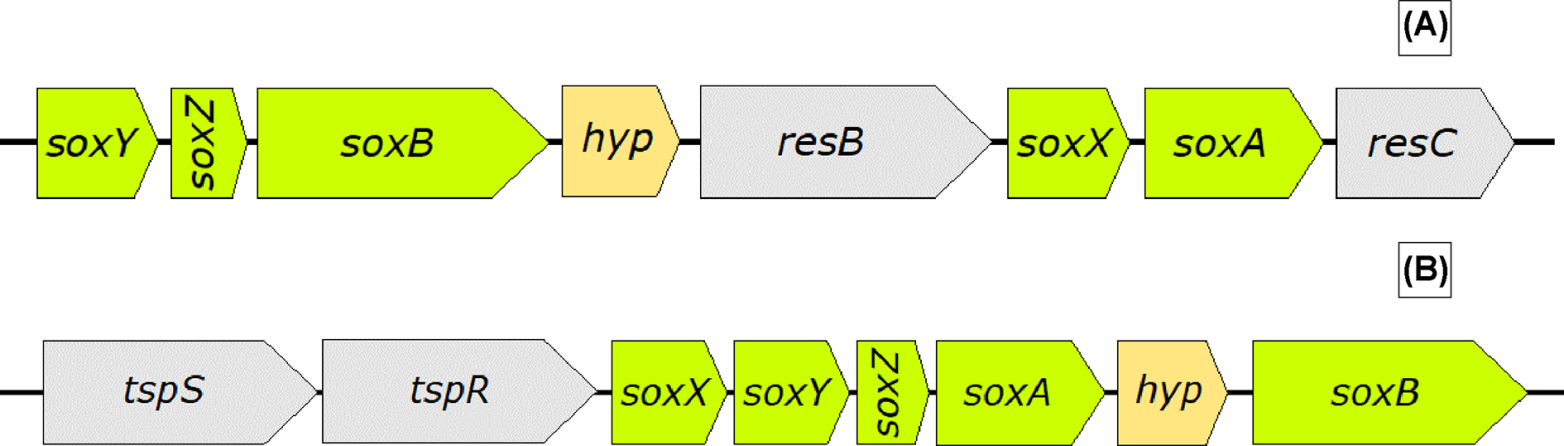
Schematic of Sox clusters present in *Acidithiobacillus* species (**A**) Sox I cluster present in *At. thiooxidans*. Adapted from [27] and (**B**) Sox II cluster present in *At. thiooxidans* and *At. ferrivorans*. Adapted from [21].

One of the key differences in the genetics underlying the sulfur metabolism of the different Acidithiobacilli species, is the presence (or absence) of genes for the Sox pathway (Text Box 1). Unlike, *At. ferrooxidans, At. thiooxidans* and *At. ferrivorans* both possess genes encoding the Sox pathway. In the case of *At. thiooxidans*, two copies have been shown to be present (Sox I and Sox II, see Figure 3A,B), whereas *At. ferrivorans* has just *sox II* [21]. The *sox* clusters in both species lack the *sox(CD)_2_* gene, and *soxX* (and *soxA* in strain SS3) has been reported to be a pseudogene in *At. ferrivorans* [26,31].

Box 1 **The Sox Pathway**

The Sox Pathway was first described in alphaproteobacterium *Paracoccus pantotrophus* [77,78], where it comprises two* c* type cytochromes, SoxXA; a covalent S-binding protein and a S compound chelating protein, SoxYZ; a dimanganese-containing protein thought to function as a sulfate thiol esterase, SoxB; and a sulfur dehydrogenase responsible for the direct oxidation of sulfite, Sox(CD)_2_ [27]. The Sox (CD)_2_ component is notably absent in many bacteria possessing the pathway, consequently forming the ‘truncated Sox pathway’ [21]. The exact mechanism of the truncated pathway is currently unclear, however, Yin et al. (2014) [27] propose that in *At. thiooxidans*, SoxXA mediates the formation of SoxYZ-S-S-SO_3_^−^ via the oxidative coupling of the sulfane sulfur from thiosulfate with a SoxY-cysteine-sulfhydryl group from SoxYZ. SoxB then catalyses the formation of SoxYZ-S-S^−^ from the SoxYZ-S-S-SO_3_^−^, with SO_3_^−^ released as sulfate. The sulfur atom of the intermediate sulfane (SoxYZ-S-S^−^) may be oxidised by SDO in the absence of Sox(CD)_2_, facilitating the formation of SoxYZ-S-SO_3_^−^, which in turn can be hydrolysed by SoxB, resulting in the regeneration of SoxYZ.

Although the sulfur oxidation pathways of the Acidithiobacilli are the most complete, there are still notable gaps in our understanding of them. For example, the enzyme responsible for sulfite oxidation in *A. ferrooxidans* and *A. ferrivorans* remains unknown, leaving a key step in the sulfur oxidation pathway of these species unresolved.

Many other species involved in bioleaching have been demonstrated to oxidise sulfur, yet models of sulfur metabolism for other acidophilic microbes are generally less complete than those of the Acidithiobacilli. However, some of the enzymes involved in RISC oxidation are shared across distinct groups. For example, a recent review demonstrates that several sulfur oxidation genes present in the Acidithiobacilli (SQR, SOR, TQO, HDR and TETH) are also present in the archaeal order Sulfolobales (which includes common bioleaching organisms such as *Sulfobacillus*, *Sulfolobus*, and *Metallosphaera*) [32]. The fact that some sulfur oxidation genes are conserved across multiple species of Bacteria and Archaea suggest that highly similar processes occur in many species capable of sulfur oxidation. Therefore, we can infer that these genes, or genes that encode proteins that perform the same function, must be present in most microbes capable of bioleaching. However, a comprehensive view of the gene products driving sulfur oxidation is still lacking for many bioleaching organisms, and significantly more work is required to fill these gaps in our understanding.

## Biochemistry of microbial iron oxidation

Ferric iron is a chemical oxidant that breaks the bonds holding sulfide minerals together [8,33]. Additionally, Fe^3+^ plays an important role in oxidising released sulfide. Therefore, the regeneration of Fe^3+^ is critical to the bioleaching process. At low pH, ferrous iron is relatively stable and abiotic oxidation is very slow [34]. Thus, microbes are vital to the iron oxidation process in acidic environments.

Acidophilic iron-oxidising microbes generate energy by reducing oxygen via electrons donated from Fe^2+^ [35]. Oxygen is the only electron acceptor that can be used in this reaction, due to the high redox potential of the Fe^2+^/Fe^3+^ couple (+0.77 at pH2) under acidic conditions [36]. As oxygen is the only freely available molecule that has a higher redox potential at low pH (+1.12V) [37], all iron oxidation mechanisms in acidophiles are aerobic.

**Figure 4:**
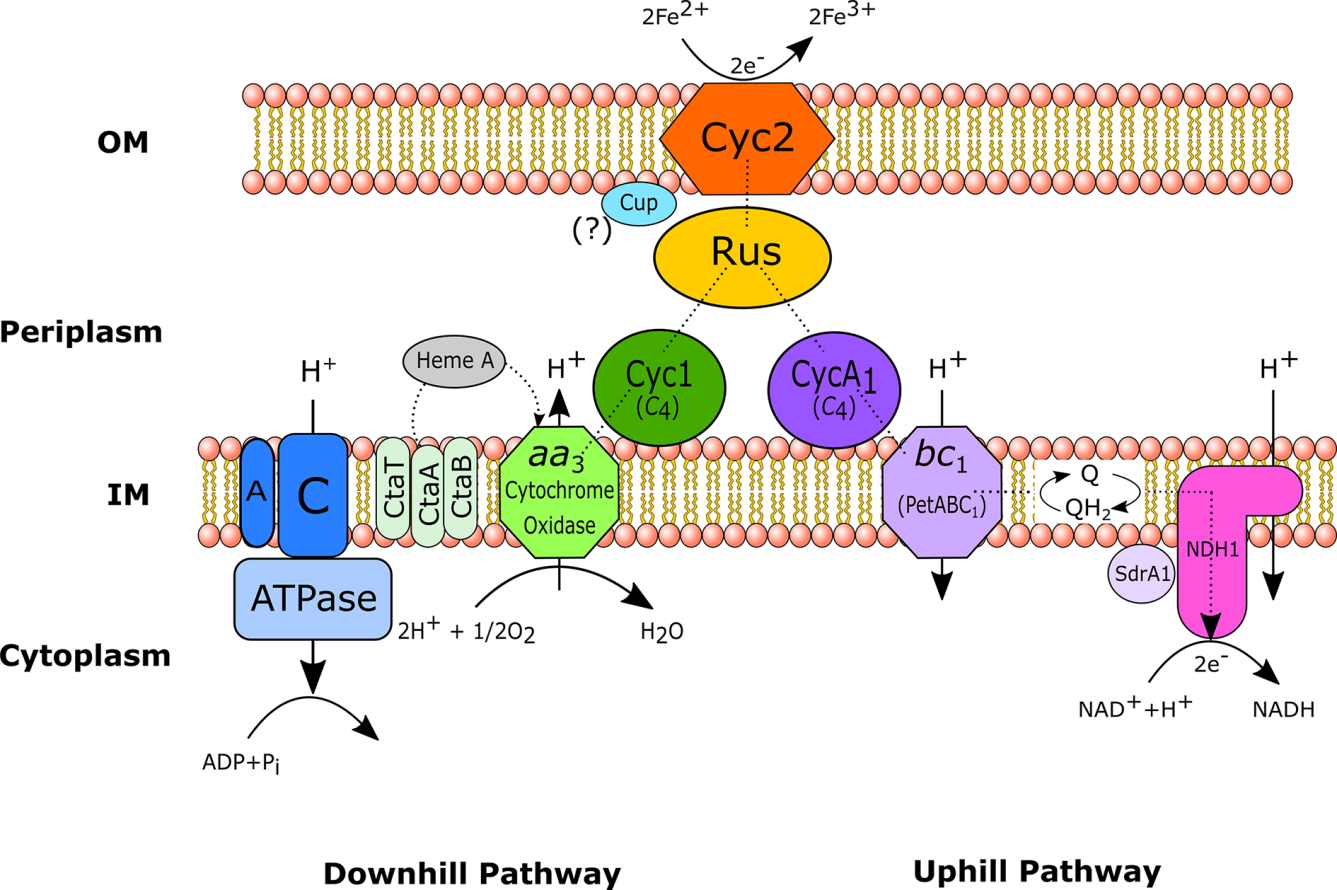
*A. ferrooxidans* ferrous iron oxidation electron transfer model The electron transport chain in *A. ferroxidans* spans the inner (IM) and outer membranes (OM), forming a super-complex that begins with a high molecular-weight outer membrane bound cytochrome *c* (*Cyc*2). Iron remains outside the cell as it is oxidised via CycC. Electrons flow from CycC to the periplasmic protein rusticyanin (Rus) and are thereafter directed to either the downhill pathway or the uphill pathway. In the downhill pathway, electrons move from Rus to the membrane-bound periplasmic cytochrome *c*, Cyc1, finally reducing oxygen via *aa*_3_-type terminal cytochrome oxidase. In the uphill pathway, electrons move from Rus to the alternate membrane-bound periplasmic cytochrome *c*, CycA_1_. From CycA_1_, electrons pass to a reverse-functioning *bc*_1_ complex positioned within the inner cell membrane and then via the membrane-associated ubiquinone pool to the NADH oxidoreductase complex (NDH1), where NAD+ is reduced. The hypothetical gene *cup* (previously ORF1), appears in the *rus* gene operon. The role of Cup is as yet undetermined, but has been speculated to include delivering copper to *aa*_*3*_and/or Rus, or facilitating electron transfer between Cyc2 and Cyc1 excluding Rus (Figure based on information and diagrams in: [17,18,34,38,39,41,44])

The nature of the acidic environment is reflected in the iron oxidation mechanism of acidophilic iron oxidisers. The high concentration of protons outside the cell combined with the neutral pH environment inside the cell membrane creates the opportunity for a trans-membrane gradient which can be exploited by acidophilic microorganisms [38]. Protons can move across the cell membrane, allowing ATP to be produced with the help of a membrane-bound ATP synthase. However, if this process were to continue in an unmitigated manner, the cytoplasm would become acidified, causing the cell to die. A counterbalance is required for the protons, in the form of negatively charged particles [34]. Ferrous iron oxidation can provide these counterbalancing electrons whilst reducing oxygen (the ‘downhill pathway’, see Figure 4).

Alongside the downhill pathway, reducing equivalents such as NADH are also produced by exploiting the electrons generated from ferrous iron oxidation. However, this process requires energy, as the NAD+/NADH couple has a significantly lower redox potential (−0.32 V) than the iron couple, meaning that if electrons are going to be moved from Fe to NAD+, they have to be pushed ‘uphill’ against the electron potential gradient. This ‘uphill pathway’ is thought to be powered by the ATP generated by the proton motive force. The downhill and uphill pathways run concurrently in iron-oxidising chemoautotrophs [34], however the uphill pathway is not required in heterotrophic iron oxidisers, as organic carbon oxidation can be used to produce reducing equivalents [39]. Although all acidophilic chemolithoautotrophs rely on both the downhill and uphill pathways working in parallel, the complexes mediating the processes notably vary between genera.

*Acidithiobacillus ferroxidans* was the first iron oxidiser to be discovered [40] and since then it has been prolifically studied. Consequently, *A. ferrooxidans* provides the most well-understood model of iron oxidation in acidophilic prokaryotes [19,41–43]. Figure 4 shows the oxidation pathway for iron in *A. ferrooxidans*.

**Figure 5:**
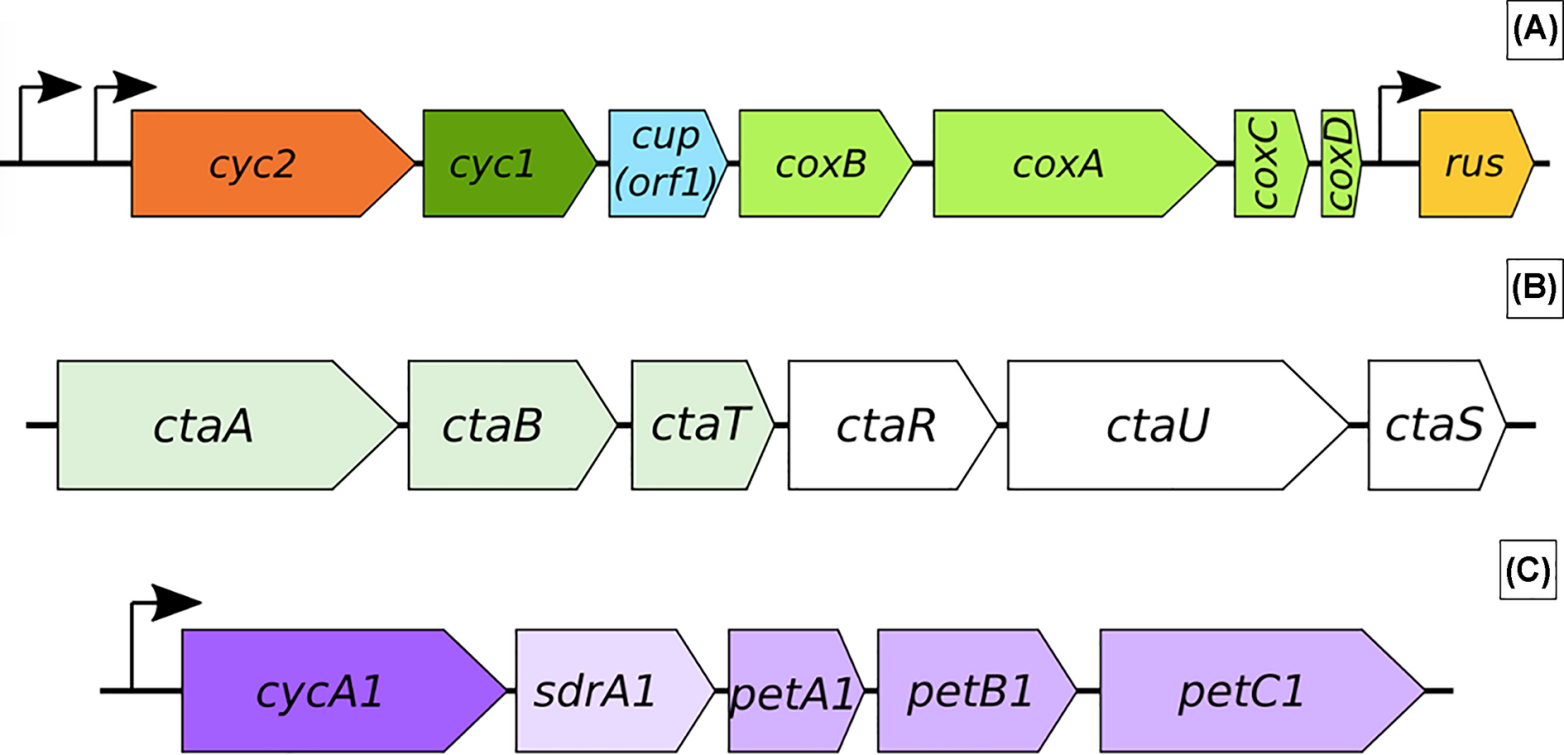
Schematics of operons associated with iron oxidation in the *Acicidithiobacilli* (**A**) The *rus* operon. Promoters are indicated by black arrows. Two promoters are present upstream of *cyc*2 which encodes the outer membrane-bound *CycC*. The* aa*_3_ subunit is encoded by *coxABCD*. Based on images and information in [23,35]. [23] (**B**) The *cta* operon. White indicates that the gene is not represented on electron transport models. Figure based on information in [19] (**C**) The* petI* operon. Promoter is shown by the black arrow [48,50].

Three operons have been identified that contain genes coding for the proteins involved in the iron oxidation pathways. The *rus* operon (Figure 5A) encodes the blue copper protein rusticyanin [45], as well as some associated cytochromes [46–48]. Downstream from *rus*, the *cta* operon (Figure 5B), contains genes associated with the biogenesis and insertion of heme* A* (*ctaABT*) which is required for *aa*_3*-*_type cytochrome oxidase activity [49]. Finally, the PetI operon (Figure 5C) encodes The *bc*_1_ complex (*petABC1*), *CycA1* (*cycA1*), short-chain dehydrogenase, SdrA1 (*sdrA1*), a NDH1 complex accessory protein, potentially serving a role as an electron shuttle [19]. This operon has been shown to be highly transcribed when *At. ferrooxidans* is grown with ferrous iron as the sole electron source [20].

Other members of the *Acidithiobacillus* genus have notable differences in their iron oxidation complexes compared to *A. ferrooxidans*. For example, in *A. ferrivorans*, two different iron oxidation pathways exist, the first is via Rus [26,51]. A second putative pathway in *A. ferrivorans* is via an iron oxidase (HIPIP: high potential iron protein) encoding gene, *iro*. Of the *Acidithiobacillus* genus, only *A. ferrivorans* and *ferriphilus* have been shown to possess *iro* [52,53]. In these species, *iro* encoded HIPIP may form the first step in the iron oxidation pathway, although complete models for this pathway are lacking to date.

**Figure 6:**
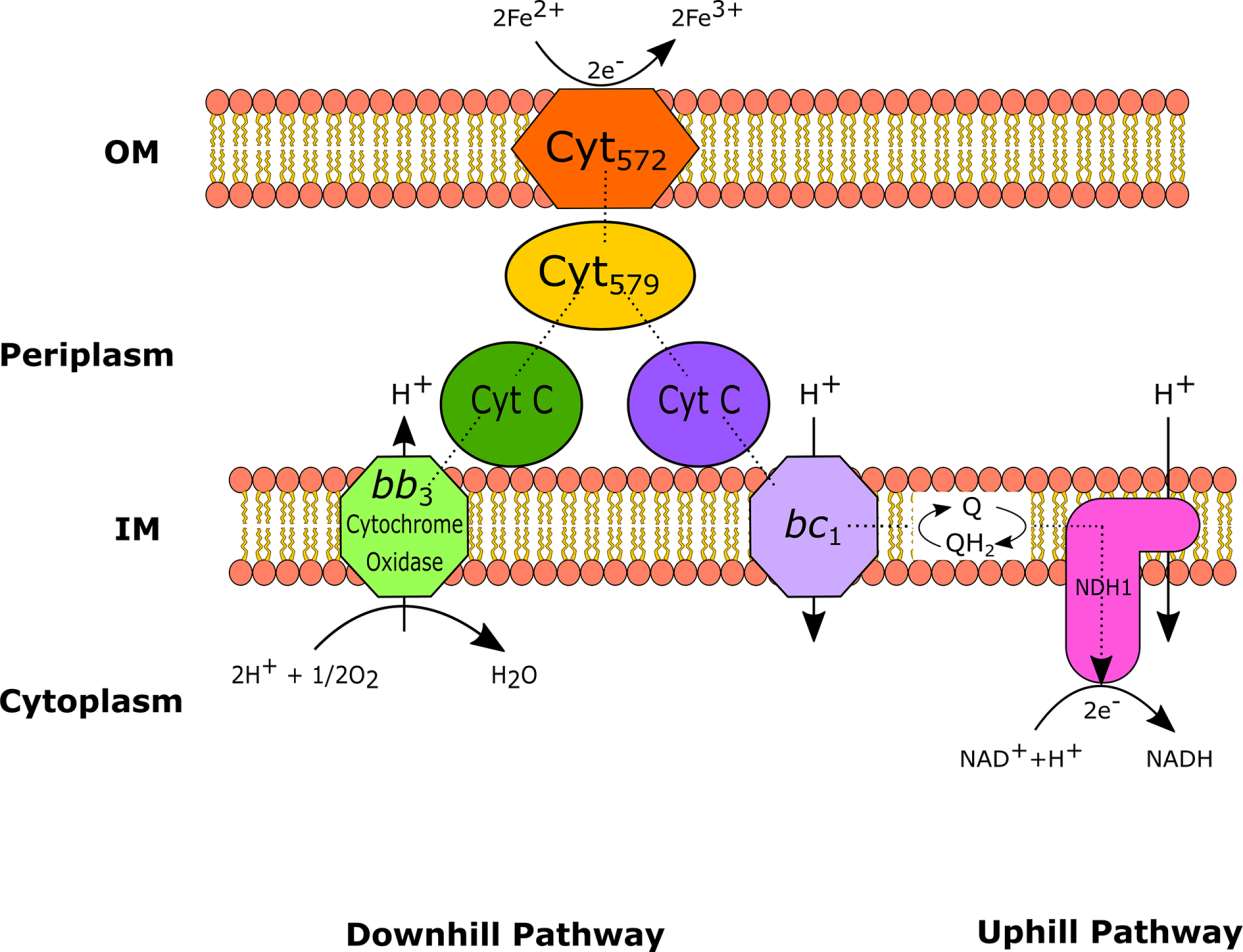
Model of iron oxidation in *L. ferrodiazotrophum* Direct oxidation occurs via the outer membrane *Cyt*_572_, with electrons passing through a potential periplasmic *Cyt*_579_ to cytochrome *c* to inner membrane bound terminal oxidases. Based on images and information in [37,58].

Iron oxidation in *Leptospirillum spp*. has been demonstrated to involve two cytochromes: *Cyt*_579_, located in the outer membrane and proposed to be the direct oxidant of iron; and *Cyt*_579_, found in the periplasm, through which electrons are passed via cytochrome *c* to a *cbb*_3_ type cytochrome oxidase [54–57]. Figure 6, shows an overview of the potential mechanism of iron oxidation in *L. ferrodiazotrophum*.

**Figure 7:**
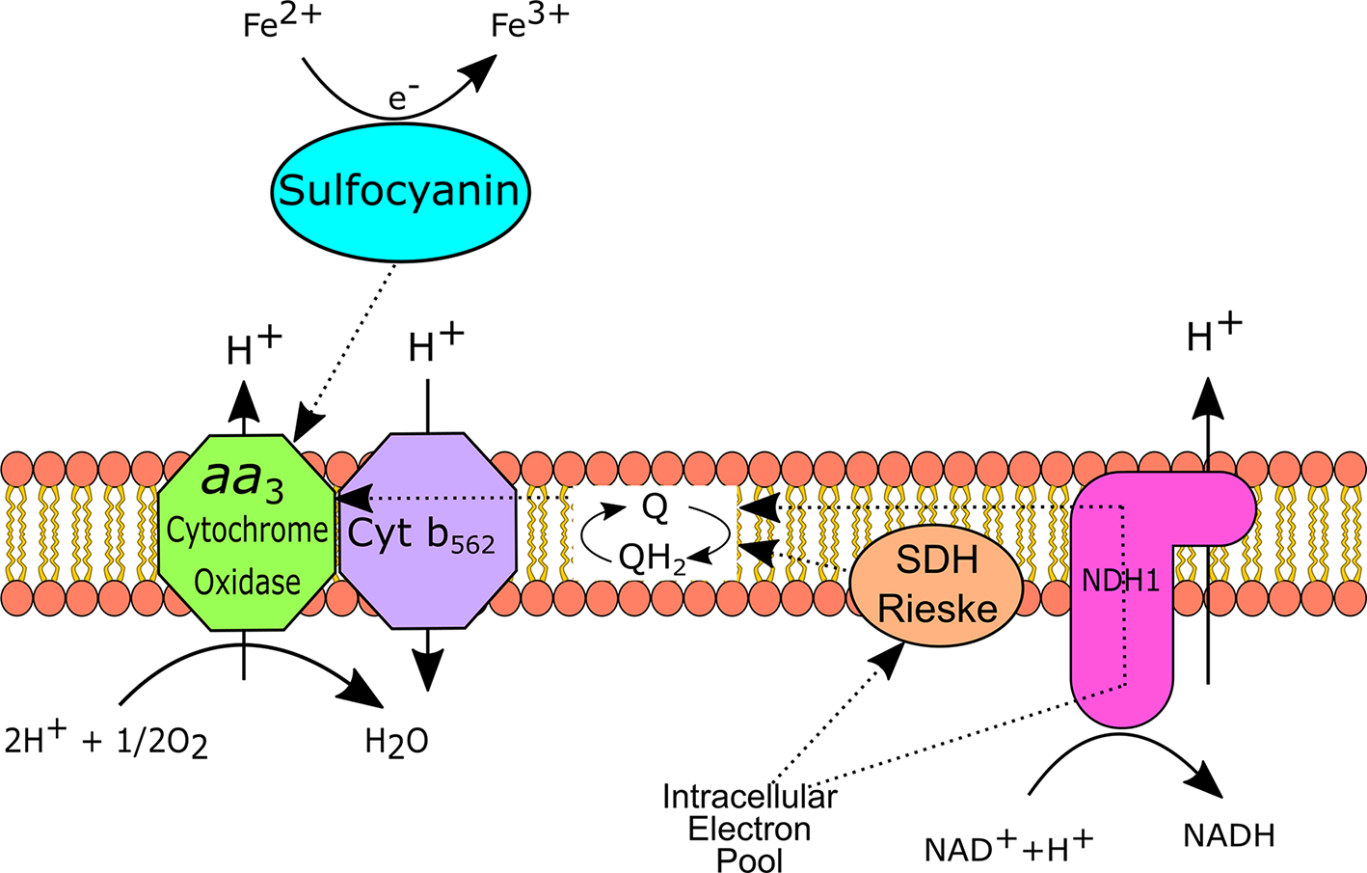
Model of iron oxidation in *F. acidarmanus* Oxidation of iron is via a sulfocyanin-type blue-copper protein, the exact location of which remains speculative. SDH: succinate dehydrogenase. Based on images and information in [37,59,60].

Iron oxidation has also been demonstrated in some acidophilic Archaea, including *Ferroplasma spp. Ferroplasma acidarmanus* is proposed to oxidise iron via a blue-copper-haem, sulfocyanin (Figure 7), the iron oxidation protein also found in the Sulfobacilli [33,59,60]. It has been speculated that the respiratory chain of both *F. acidarmanus* and *F*. Type II contains a super-complex, which consists of a Rieske-cytochrome *bc*_1_ complex and terminal oxidases [59].

A number of gaps remain in our knowledge with regards to the mechanisms involved in prokaryotic iron oxidation; the pathways and associated genes involved in iron oxidation have thus far only been identified in a handful of species. For almost all common bioleaching organisms, the mechanism of iron oxidation remains at least partially unknown.

## Mechanisms of bioleaching: the thiosulfate and polysulfide pathways

**Figure 8:**
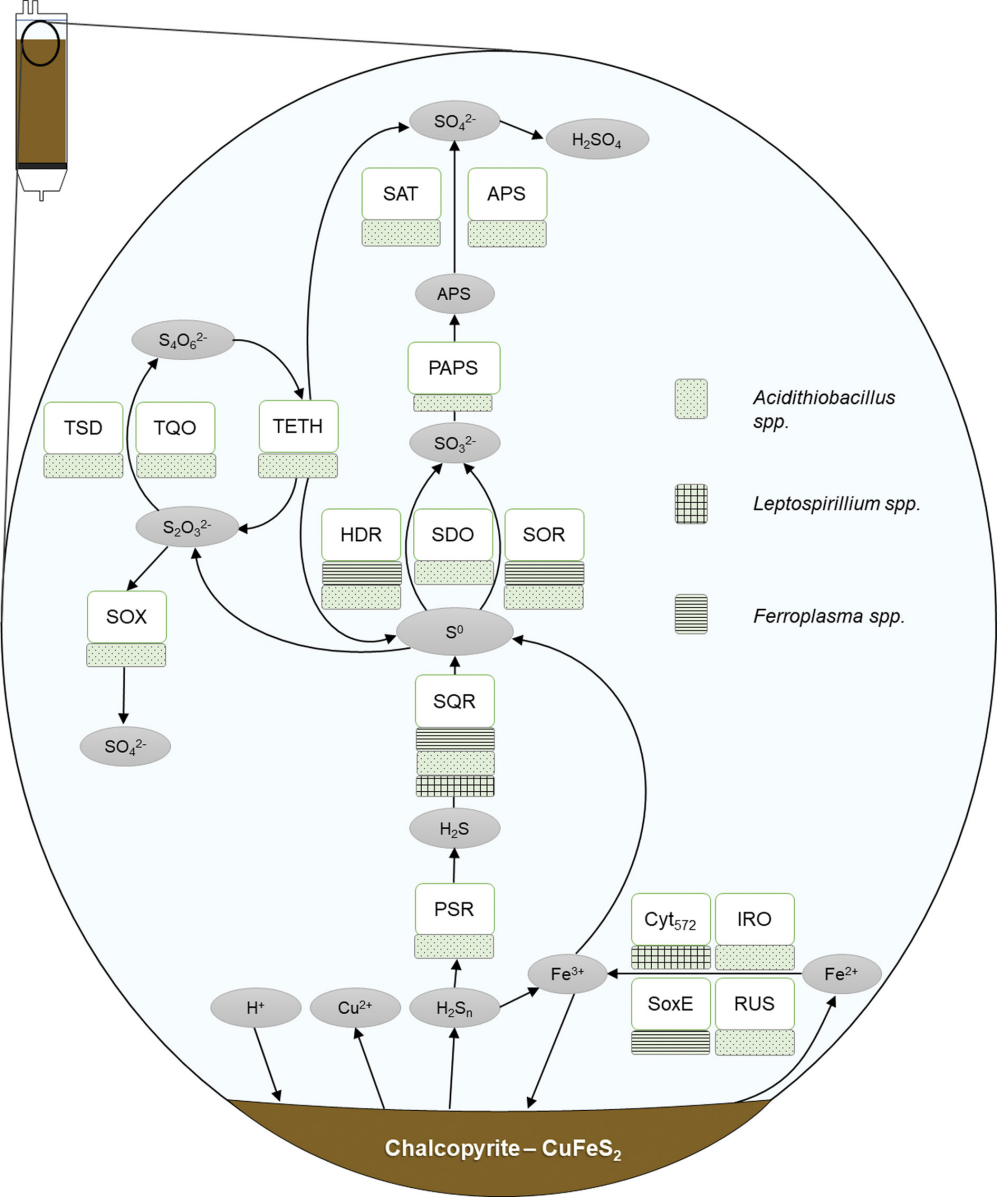
Model of chalcopyrite dissolution via the polysulfide pathway PSR: polysulfide reductase, SQR: sulfide-quinone reductase, SOR: sulfur oxygenase reductase, SDO: sulfur dioxygenase, HDR: heterodisulfide reductase, SAT: sulfate adenylyltransferase, TetH: tetrathionate hydrolase, TQO: thiosulfate-quinone oxidoreductase, TSD: thiosulfate dehydrogenase, SOX: sulfur oxidation pathway, RUS: rusticyanin, SoxE: Sulfocyanin, IRO: high potential iron-sulfur protein, *Cyt*_579_: Cytochrome 572.

**Figure 9:**
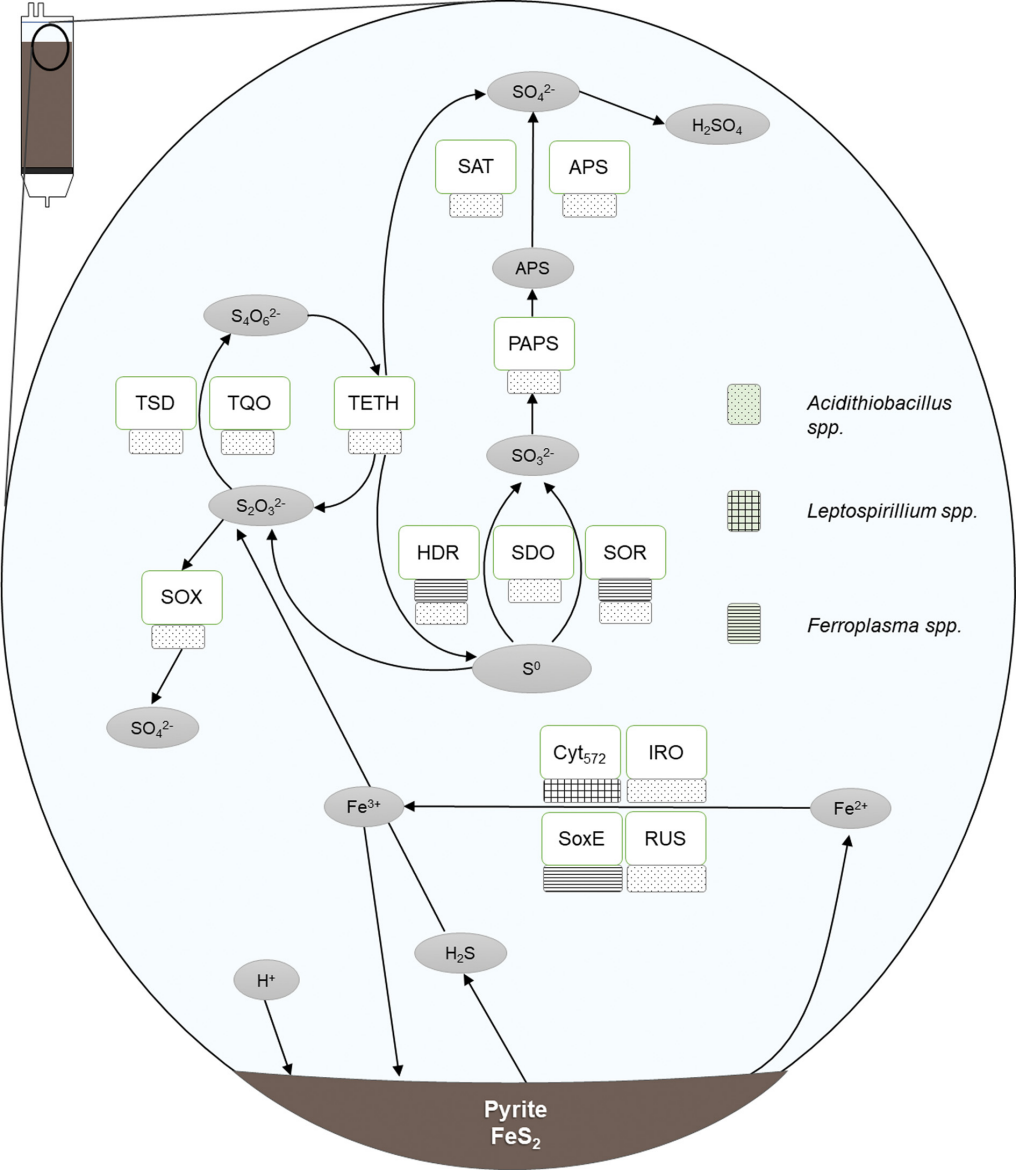
Model of pyrite dissolution via the thiosulfate pathway SQR: sulfide-quinone reductase, SOR: sulfur oxygenase reductase, SDO: sulfur dioxygenase, HDR: heterodisulfide reductase, SAT: sulfate adenylyltransferase, TetH: tetrathionate hydrolase, TQO: thiosulfate-quinone oxidoreductase, TSD: thiosulfate dehydrogenase, SOX: sulfur oxidation pathway, RUS: rusticyanin, SoxE: Sulfocyanin, IRO: high potential iron-sulfur protein, *Cyt*_579_: Cytochrome 572.

The mechanism by which sulfide minerals are oxidised by microbes varies depending on the mineral properties. The two pathways are the ‘polysulfide pathway’ and the ‘thiosulfate pathway,’ so named due to the intermediate sulfur species generated during mineral dissolution [61]. Acid-insoluble minerals, such as pyrite and tungstenite are oxidised via the thiosulfate pathway, while the polysulfide pathway is the mechanism by which acid–soluble minerals such as chalcopyrite, galena and arsenopyrite are broken down (Figures 8 and 9). The configuration of electrons in a particular sulfide mineral determines whether or not it is acid–soluble. In the acid–soluble group, valence bands are obtained from the orbitals of both the sulfur and metal atoms. Therefore, in these minerals, protons can cleave the bonds between sulfide and mineral by oxidising electrons in the valence band. In the acid–insoluble group, valence bands are derived from the orbitals of metal atoms only. Consequently, valence bands do not contribute to bonding between the sulfide and the metal(s), leaving them resistant to proton attack [10,61,62]

The polysulfide pathway involves minerals whose metal-sulfur bonds can be broken prior to sulfur oxidation. Consequently, these minerals are susceptible to proton attack, which is the initial stage of mineral breakdown. In acidic conditions, protons facilitate the cleaving of metal from the sulfur moiety and the subsequent formation of hydrogen sulfide. However, in the presence of ferric iron, a sulfide cation (H_2_S^+^) is formed (in place of H_2_S) which spontaneously dimerises, leaving H_2_S_2_. This is subsequently oxidised by ferric iron to elemental sulfur, via additional polysulfides (eqn 1). Alternatively, this conversion may be enzymatically facilitated. Where this step is microbially mediated, it occurs in a stepwise manner. The first step may be catalysed by PSR in *A. ferrooxidans* [63]. Hydrogen sulfide is then oxidised to elemental sulfur via SQR (eqn 2). Finally, elemental sulfur may be microbially oxidised to sulfate, then sulfuric acid (eqn 3) [13,61]. Again, this is a stepwise conversion, with elemental sulfur converted to sulfite via HDR, SDO or SOR.

(1) $$\text{MS}+\;{\text{Fe}}^{3+}+{\text{H}}^{+}\text{→}\;{\text{M}}^{2+}+0.5\;{\text{H}}_{2}{\text{S}}_{n}\;+\;{\text{Fe}}^{2+}\;(\text{where}\;n\;\geq \;2)$$

(2) $$0.5\;{\text{H}}_{2}{\text{S}}_{n}\;+\;{\text{Fe}}^{3+}\text{→}\;0.125\;{\text{S}}_{8}\;+\;{\text{Fe}}^{2+}+\;{\text{H}}^{+}$$

(3) $$0.125\;{\text{S}}_{8}\;+\;1.5\;{\text{O}}_{2}\;+\;{\text{H}}_{2}\text{O}\;\to \;{\text{SO}}_{4}^{2-}\;+\;2\;{\text{H}}^{+}$$

The mechanism of sulfite oxidation remains elusive for many common species; however, there is a proposed pathway in *A. thiooxidans* via the enzyme PAPS. The subsequent stage of APS oxidation to sulfate is facilitated by SAT or APS enzyme. In species possessing a Sox pathway (a multi-enzyme sulfur oxidation system, see Text Box 1), many of the preceding sulfur oxidation reactions may be facilitated by it, as this system is capable of oxidising sulfide, elemental sulfur, sulfite, and thiosulfate, producing a final product of sulfate.

Alternatively, elemental sulfur may be converted to thiosulfate abiotically or by SOR [64]. Thiosulfate is oxidised to tetrathionate via TQO and TSD. TETH can then disproportionate tetrathionate to thiosulfate, elemental sulfur and sulfate. Thiosulfate may also chemically decompose to tetrathionate, sulfur or sulfite [65].

As sulfate forms sulfuric acid when dissolved in water, protons are regenerated to serve as an oxidant by which acid-soluble sulfide minerals (e.g., chalcopyrite) are further broken down, aided by the ferric iron regenerated by the iron-oxidising organisms. Reduced (ferrous) iron released from the mineral is oxidised to ferric iron via a number of potential pathways (Rus, SoxE, Iro, *Cyt*_572_).

For acid insoluble minerals, such as pyrite, protons do not initiate breakdown. Instead, bioleaching proceeds via the thiosulfate pathway, in which thiosulfate is generated via ferric iron hexahydrate oxidation of the mineral and subsequent cleaving of (eqn 4) the Fe-S_2_ bond [66]:

(4) $${\text{FeS}}_{2}\;+\;6\;{\text{Fe}}^{3+}\;+\;3\;{\text{H}}_{2}\text{O}\to \;{\text{S}}_{2}{\text{O}}_{3}^{2-}\;+\;7\;{\text{Fe}}^{2+}\;+\;6\;{\text{H}}^{+}$$

This thiosulfate is rapidly oxidised, either abiotically with ferric iron, or via TQO or TSD in sulfur-oxidising microbes. The oxidation of thiosulfate results in tetrathionate, which may then degrade to one of several compounds, including: trithionate, pentathionate, sulfite and elemental sulfur, although the quantity of these products is limited compared to sulfate [62,67]. Indeed, these species may then be oxidised to sulfate via biotic or abiotic reactions.

The thiosulfate to sulfate stage of the process can be summarised in (eqn 5) [10]:

(5) $${\text{S}}_{2}{\text{O}}_{3}^{2-}\;+\;8\;{\text{Fe}}^{3+}\;+\;5\;{\text{H}}_{2}\text{O}\;\to \;2\;{\text{SO}}_{4}^{2-}\;+\;8\;{\text{Fe}}^{2+}\;+\;10\;{\text{H}}^{+}$$

## Omic and meta-omic studies of iron- and sulfur-oxidising species

Omics (e.g. genomics, transcriptomics and proteomics) are approaches to studying organisms, i.e. through sequencing and bioinformatic analyses of nucleic acids or proteins. These approaches can help us gain new insights into the bioleaching potential of as-yet uncultured species, or organisms that are recalcitrant to transformation through genetic manipulation. For example, genomic analysis was essential to the production of a model of iron oxidation in *Ferrovum*, as the organism was contaminated with a strain of *Acidiphilium* [68]. Additionally, omics studies can leverage existing datasets to gain new functional information on microbial metabolism and function. For *Acidiferrobacter* spp, comparative genomics of available genomic sequences was used to reconstruct iron and sulfur oxidation pathways [69].

Omics techniques can be scaled up to include analyses of whole microbial communities, i.e. ‘meta-omics’. The benefits of a ‘meta-’ approach include the absence of the requirement to obtain a pure culture of one species, which can lead to the identification of sulfur and iron oxidation mechanisms in species that could otherwise not be studied. Meta-omic studies have identified the presence of sulfur and iron oxidation associated genes in many non-isolated organisms, including strains of *Leptospirillum* [58,70], *Rhodanobacter*-like and* Thiomonas*-like populations [71], a *Ferroplasma-*like population [72], and a member of the family *Gallionellaceae* [73].

The advent of meta-omics has allowed several researchers to gain otherwise unobtainable information on the mechanisms of sulfur and iron oxidation at a community level. For example, metagenomic and metatranscriptomic techniques have been used to study the abundance and gene expression of all community members *in situ* during bioleaching with a bioleaching consortium that contained non-isolated species [63]. Using this approach allowed the creation of community-level models of sulfide mineral dissolution pathways. Additionally, this work provided the first evidence of sulfur oxidation gene expression in the Archaeon *Cuniculiplasma divulgatum*. Meta-omics can similarly be used to examine community dynamics in artificially created bioleaching consortia (e.g. [74]). Meta-omics can therefore be regarded as a crucial tool in future developments in bioleaching, as understanding in situ interactions is key to developing biotechnological applications of a microbial community [75,76].

## Conclusions and future directions

Bioleaching exploits the metabolisms of acidophilic microbes to break down sulfide minerals. This technique is increasingly being explored as a low-input, low-cost way of extracting metals from low-grade ores. In this review, we have shown that the mechanisms underlying bioleaching are complex, with numerous enzymes required to facilitate the multi-step sulfur and iron oxidation processes. The two pathways of breakdown associated with different types of sulfide mineral were also highlighted, with currently known enzymes incorporated into up-to-date models of bioleaching pathways.

There are still many gaps in our understanding of the mechanisms underlying bioleaching, especially the enzymes involved in sulfur metabolism. Modern research techniques, such as high-throughput sequencing and bioinformatics can help us to continue to elucidate these metabolic pathways and identify genes associated with bioleaching in novel species. In turn, this information could help us to optimise selection of organisms for bioleaching, as well as identifying novel uses for acidophiles.

## Summary

- Bioleaching uses the iron- and sulfur-oxidising metabolisms of acidophilic microorganisms to extract metals from ore.
- Sulfur oxidation is a complex, multi-step process that is facilitated by a large number of enzymes, as demonstrated by the most complete model of sulfur oxidation in the extremophile *A. ferrooxidans.*
- Modern omics and meta-omics techniques offer new opportunities to resolve gaps in our understanding of bioleaching mechanisms, as well as offering the potential to optimise the selection of organisms for bioleaching applications.

## Open Access

Open access for this article was enabled by the participation of University College London in an all-inclusive *Read & Publish* agreement with Portland Press and the Biochemical Society under a transformative agreement with JISC.

## Abbreviations

HDR: heterodisulfide reductase
RUS: rusticyanin
SAT: sulfate adenylyltransferase
SDO: sulfur dioxygenase
SOR: sulfur oxygenase reductase
SOX: sulfur oxidation pathway
SQR: sulfide-quinone reductase
TetH: tetrathionate hydrolase
TSD: thiosulfate dehydrogenase
TQO: thiosulfate-quinone oxidoreductase
